# A Comparison of Simulation-Based Education and Problem-Based Learning in Pre-Clinical Medical Undergraduates

**DOI:** 10.15694/mep.2020.000172.1

**Published:** 2020-08-19

**Authors:** Tyler Larsen, Nicholas J. Jackson, Jason Napolitano

**Affiliations:** 1 VA Greater Los Angeles Healthcare System; David Geffen School of Medicine at UCLA; 2 David Geffen School of Medicine at UCLA

**Keywords:** High-Fidelity Simulation, Simulation-Based Medical Education, Problem-Based Learning, Preclinical Undergraduate Medical Education

## Abstract

This article was migrated. The article was marked as recommended.

**Introduction:** Despite the increasing popularity of high-fidelity simulation (SIM), few studies compare the effectiveness of SIM with established small-group modalities such as problem-based learning (PBL) for factual learning among pre-clinical medical undergraduates.

**Methods:** 162 second-year medical students were randomized to two groups. The first group encountered teaching points related to the management of hypertensive emergency during a traditional PBL session, while the second group covered the same teaching points through SIM. Both groups then completed a post-test. The students were subsequently crossed-over to the opposite modality and assessed on teaching points related to the management of chronic obstructive pulmonary disease exacerbations.

**Results:** A total of 162 students participated in the study. For the hypertension case, the average proportion of correct questions for the SIM intervention was 83% compared to 70% for the PBL intervention (OR = 3.1, 95% CI [1.3, 7.2], *p*=0.009). For the COPD case, the average proportion of correct questions for the SIM intervention was 73% compared to 51% for the PBL intervention (OR = 2.6, 95% CI [1.8, 3.8], *p* < 0.001).

**Conclusion:**Students scored significantly higher with SIM in both cases, indicating that SIM may be superior to PBL for factual learning among students with limited clinical exposure.

## Introduction

Since its inception nearly a half-century ago, problem-based learning (PBL) remains a staple of small-group medical education ( Kinkade, 2005; Hartling *et al.*, 2010; Poulton *et al.*, 2014). Originally developed to foster the ability “to evaluate and manage patients with medical problems effectively, efficiently, and humanely”, PBL also allows students to identify areas for continued exploration and lifelong learning ( Barrows and Tamblyn, 1980; Neville and Norman, 2007; Neville, 2009; Norman, 2012). With the advent of new and more readily accessible technologies, however, the use of simulation-based medical education (SBME) has become an increasingly popular small-group educational modality across all spheres of health education, including graduate and undergraduate medical education, nursing education, and pharmacy education ( Seybert *et al.*, 2012; Cohen *et al.*, 2013; Lopreiato and Sawyer, 2015; Shin, Park and Kim, 2015; Alluri *et al.*, 2016).

As the utilization of SBME modalities has increased, so too has the evidence for the efficacy of SBME. Within the realm of graduate medical education alone, SBME has been shown to improve trainee performance in a variety of procedural and interpersonal tasks across a broad range of disciplines, including colonoscopy, central venous catheter insertion, laparoscopic surgical procedures, and ethical dilemmas ( McGaghie *et al.*, 2011; Barsuk *et al.*, 2012; Bearman *et al.*, 2012; Smith *et al.*, 2012; Cohen *et al.*, 2013; Schmidt *et al.*, 2013; Singer *et al.*, 2013). SBME has also become increasingly popular in the realm of undergraduate medical education, but studies on the efficacy of simulation to date have largely been limited to students at the clinical level (traditionally third and fourth year students who are completing clinical clerkships) ( Ten-Eyck, Tews and Ballester, 2009; Ko *et al.*, 2011; Littlewood *et al.*, 2013). Results from these studies have mostly validated the use of SBME as an effective teaching modality in this population, but some results have been equivocal ( Wenk *et al.*, 2009; Kerr *et al.*, 2013). High-fidelity patient simulation (SIM) in particular is increasingly utilized in the context of pre-clinical undergraduate medical education, but its efficacy as it compares to traditional teaching modalities of problem-based and didactic-based education is just now being elucidated ( Harris, Ryan and Rabuck, 2012; Littlewood *et al.*, 2013; Alluri *et al.*, 2016).

Despite the growing popularity of SBME, there is limited data to quantify how SBME compares in efficacy to the other small-group educational modalities with which it directly competes, namely PBL, for knowledge acquisition ( Alluri *et al.*, 2016). Several studies have demonstrated a significant improvement in factual learning with SIM as compared to paper-based PBL, but these studies have been limited to advanced medical students at the clinical level ( Steadman *et al.*, 2006; Schwartz *et al.*, 2007). Therefore, the purpose of this study is to compare small group high-fidelity simulation with PBL for factual learning in pre-clinical medical students.

## Methods

### Population Setting

All second-year (preclinical) medical students enrolled in the 2014-2015 academic year of the Cardiovascular, Renal, and Respiratory Medicine II (HBD409) course at the David Geffen School of Medicine at UCLA were included in this randomized, controlled cross-over study conducted within the parameters of an established problem-based learning curriculum. Student participation was voluntary through their enrollment in the course. All study activities including completion of multiple choice self-assessment questions to assess learning were conducted within the context of the course and no extracurricular activities were required. UCLA Human Subjects Protection Committee approved exemption from full IRB review for this study as all data were de-identified and analyzed after all students had completed and received a final grade for the course. It should be noted that this study was separate from a previous study at our institution by one author (JN) and involved a different cohort of students and separate IRB approval ( Alluri *et al.*, 2016).

### Study Design

At the beginning of the course, 162 second-year medical students were divided into PBL groups of 8-9 students, as is the routine with every course at our medical school. This made 20 groups, 10 of which were randomly assigned to group A and 10 of which were assigned to group B. The traditional PBL curriculum requires a two-hour meeting at the start of each week and a two-hour meeting for the resolution of the patient case on the last day of each week. The teaching sessions analyzed in our study took place during this second meeting of both the third and sixth weeks of the course. During the third week of the course, group A encountered structured teaching points related to the diagnosis and management of hypertensive emergency in the traditional PBL format. A specific addendum to the existing PBL patient case was utilized for this purpose. Group B encountered identical structured teaching points related to an identical case in the format of a 10-minute SIM with subsequent debriefing session. After the completion of their respective modalities, all students completed three written multiple-choice questions to assess their learning of the teaching points.

During the sixth week of the course, the students were crossed over to the opposite modality. During this week, Group B encountered structured teaching points related to the management of a chronic obstructive pulmonary disease (COPD) exacerbation in a traditional PBL format while Group A completed an identical SIM case with identical teaching points. All students again individually completed three written multiple-choice questions as a post-test to assess the learning of the teaching points. Each post-test was scored from 0 to 3 via a web-based course-management system. All PBL sessions were completed as part of a stable group (the students and tutors remained in the same group for all eight weeks of the course). Two faculty members with experience conducting and debriefing simulation sessions proctored all the simulation sessions.

### Simulator Sessions

The SIM sessions were conducted in the same manner as other sessions at our institution ( Alluri *et al.*, 2016). As in previous studies, a METI (Medical Education Technologies, Sarasota, FL) high-fidelity mannequin was used for all SIM components of this study ( Alluri *et al.*, 2016). All students were familiar with the simulator system through their prior participation in simulator sessions as a part of their preclinical curriculum.

Each SIM scenario in this study lasted ten minutes. At the start of each session the students were introduced to the patient’s chief complaint, brief history, and initial vital signs by the faculty proctor. Students were then able to obtain further history by interviewing the mannequin and information was provided by a simulation technician or the faculty proctor according to a written simulation script. The students were also able to perform examination maneuvers, request clinical data, and administer therapeutic interventions such as medications. A telemetry monitor provided real-time physiologic data according to the case script and student interventions. Faculty proctors participated in the simulation in the role of a bedside nurse and were present throughout the entire session.

Upon completion of the simulation all students received a ten-minute debriefing session led by the faculty proctor. During the debriefing session proctors emphasized pre-determined teaching points which were identical to the teaching points found in the tutor notes for instructors leading the traditional PBL sessions. A single proctor ran each SIM session with a simulation specialist in the room controlling the mannequin. Simulations were run in two rooms simultaneously with a new group of 8-9 students coming into each simulation room every 20 minutes.

### Problem-Based Learning Sessions

PBL sessions were conducted according to the existing PBL curricular framework at our institution. All PBL sessions for this study were conducted on the second meeting of the problem-based learning component for each respective week. A specific case addendum was written to existing paper-based PBL cases. Each addendum consisted of one page added to the existing handouts in which students were given a patient encounter identical to the simulation case. Students were encouraged to explore how they would manage the patient through small-group discussion. All PBL faculty facilitators were given specific instructions to reinforce the teaching points related to the management of the given presentation prior to moving forward with the next section of the PBL case.

### Post-Test Assessment

All students were individually assessed on their learning of the predetermined teaching points through a total of six written multiple-choice post-test assessment questions- three for each case. Upon completion of each case the students encountered three multiple-choice questions incorporated into the existing weekly self-assessment framework for the course. These weekly self-assessments were administered through a web-based course-management system and completed at a time of the student’s choosing between 5:00PM on the last day of the week and 8:00AM on the first day of the following week. These assessments were open-book format, although students were encouraged to complete these assessments on their own and without assistance from outside resources. All assessments were scored by the course management software according to an answer key written prior to administration and each student received an individual score 0 through 3 for each post-test. All student scores were de-identified and all students were randomly assigned a number through 1 through 162 for the purposes of examining the effect of the crossover intervention. Student performance on the assessment did not impact their final grade for the course.

### Data Analysis

The average number of questions correct were calculated for each post-test and compared between the PBL and SIM groups for both the hypertension and COPD cases. Additionally, a mixed effects logistic regression model was used to examine between subject differences in educational intervention (SIM vs PBL) at each week and to examine between subject differences in test ordering (Week 6 vs Week 3). Within-subject differences in the educational intervention and in test ordering were also examined using data combined from both weeks and both interventions respectively. A p-value of <0.05 was considered statistically significant. All analyses were conducted in Stata Version 13, StataCorp LP (College Station, Texas).

## Results/Analysis

**Figure 1.**
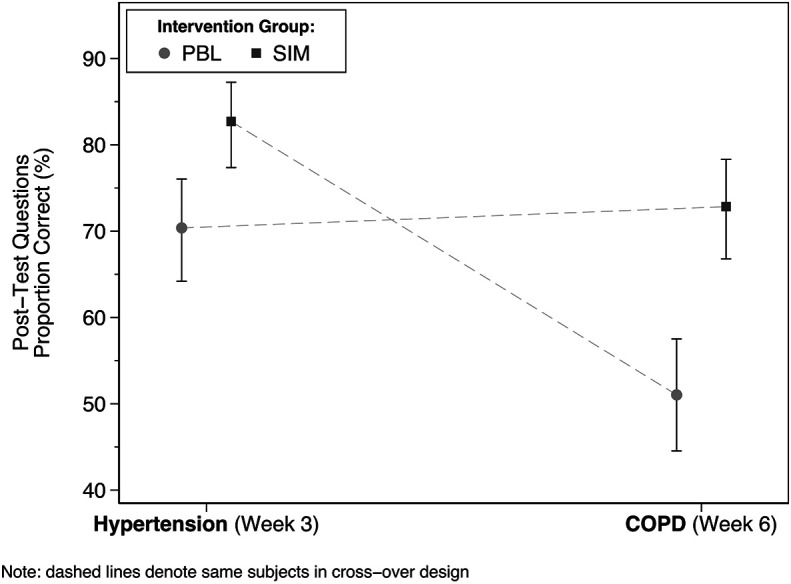
Post-Test Proportion Correct by week.

A total of 162 students were studied. Eighty-one students were assigned to group A, which completed the PBL intervention for the hypertensive emergency case and the SIM intervention for the COPD exacerbation case. The other eighty-one students (Group B) completed SIM for the hypertensive emergency case and PBL for the COPD case. All students completed both post-tests except for one student in Group B who did not complete the COPD post-test. In both cases, the students who completed SIM scored significantly higher on the post-test questions than those who completed PBL ( Table 1). For the hypertension case (week 3), the average proportion of post-test questions correct across students completing the SIM intervention was 83% compared to an average post-test score of 70% for students completing the PBL intervention (odds ratio = 3.1, 95% CI [1.3, 7.2], *p*=0.009). For the COPD exacerbation case (week 6), the average proportion of post-test questions correct across students completing the SIM intervention was 73% compared to an average post-test score of 51% for students completing the PBL intervention (odds ratio = 2.6, 95% CI [1.8, 3.8], *p* < 0.001). For both the PBL and SIM groups, the combined post-test scores for the hypertension case (86% in week 3) were significantly higher ( *p* < 0.001) than for the COPD case (62% in week 6). A description of the students’ correct responses to the post-test questions is provided in Table 1 and a graphical depiction is provided in Figure 1.

**Table 1.** Comparison of Proportion correct between PBL and SIM with 95% Confidence Intervals

	PBL Group **% Correct**	SIM Group **% Correct**	Odds Ratio **SIM vs PBL**	p-value
**Hypertension Case** **(Week 3)**	70 (64, 75)	83 (77, 87)	3.1 (1.3, 7.2)	0.009
**COPD Case** **(Week 6)**	51 (45, 58)	73 (67, 78)	2.6 (1.8, 3.8)	<0.001

Given that the study permitted a cross-over analysis with repeated within-subject measurements, a mixed effects logistic regression model was used to determine if the effects of the educational intervention (SIM vs PBL) differed depending on the type of clinical scenario (hypertension in week 3 vs COPD in week 6). Modeling the interaction of week by intervention yielded a p-value of 0.76, indicating no significant difference in the performance of the two simulation groups in their respective weeks. Thus, the differences between the two interventions (SIM vs. PBL) did not differ between the COPD and hypertension cases.

## Discussion

This study demonstrated a significant improvement in factual learning and knowledge acquisition in students who completed high-fidelity simulation training compared with problem-based learning. In fact, when looking at both weeks of the study, the odds of answering the post-test questions correctly after having completed SIM were over three times more likely than they were after completing PBL. These results also demonstrate that post-test performance was improved with simulation regardless of the week in which the students received the simulation intervention. After completing the analysis, it was apparent that the questions in week 6 were more difficult than in week 3, but ultimately there was no other significant difference between the two weeks. This also demonstrates that ordering of the interventions played no significant role and that it did not matter whether the students performed SIM or PBL first. This study, to our knowledge, is one of the largest randomized studies comparing high-fidelity simulation with problem-based learning for the purposes of knowledge acquisition at the preclinical level. Previous studies in clinical-level medical students have shown similar improvements in post-test scores with simulation as compared to PBL ( Schwartz *et al.*, 2007).

We chose to compare SIM to PBL as both modalities are interactive small group activities that revolve around working through a patient case. PBL is pervasive in modern medical education, with greater than 70% of medical schools in 2005 reporting the use of PBL in some capacity in the preclinical years ( Kinkade, 2005; Wood, 2008). Yet, despite the ubiquity of PBL, the data surrounding its efficacy and outcomes as compared with traditional pedagogical methods remains mixed ( Koh *et al.*, 2008; Okuda *et al.*, 2009). While PBL has been shown to improve abilities such as clinical reasoning ( Neville and Norman, 2007; Neville, 2009; Norman, 2012), PBL has not proved to be a universally superior curricular modality ( Steadman *et al.*, 2006; Koh *et al.*, 2008; Wenk *et al.*, 2009). Intuitively this is logical, as no one educational intervention or pedagogical method is likely to perfectly encompass all the nuanced aspects of medical education. Thus, it is fair and prudent, given the pervasive use of PBL and its widespread endorsement within the medical education community, to compare it as a small group teaching modality with simulation.

While both PBL and SIM are small-group modalities, a key difference is that the high-fidelity simulator uses physical equipment and technology that provides students with psychomotor stimuli and real-time feedback. The SIM milieu may evoke greater emotional engagement and concentration than a classroom-based PBL session. This study corroborates support for the hypothesis posited by others that such visual, auditory, and tactile stimuli engage the learner beyond the purely cognitive aspects of PBL ( Issenberg *et al.*, 2005). Importantly, SIM provides the learner with a safe environment in which to make decisions and analyze the consequences of those decisions ( Issenberg *et al.*, 1999; Wood, 2008). Such engagement may reinforce learning concepts beyond oral discussion of a written case.

Our study has some limitations. First, some may contend that PBL was not designed to optimize factual learning. We, however, would assert that most PBL cases and tutor guides have specific learning points written into them. Therefore, it is often expected that students will assimilate new knowledge during PBL sessions alongside the opportunities to ask and answer clinical questions and to strengthen communication and teamwork skills. That stated, we are not arguing that SIM should replace all PBL. However, the activation of the SIM lab may make certain facts “stickier” and therefore more easily learned by students. More work remains to be done to determine which concepts would be better served through PBL and which would be better served through simulation.

Second, despite our sessions taking place in the “preclinical” years, our learning points for this study focused on a combination of patient management and basic science principles, rather than purely focusing on basic science. Additionally, all of our PBL groups and SIM sessions had one or more clinicians as a facilitator. Our approach might not yield the same results for medical schools not using faculty with clinical experience to facilitate their sessions. Similarly, the high-fidelity simulators used in our study are expensive and the SIM sessions require intensive investment of time by faculty. Faculty at other medical schools might wonder whether it is worth the investment in equipment and faculty to incorporate high-fidelity simulation into the first one or two years of medical school to obtain a moderate improvement in immediate recall of teaching points. Future research might look at whether similar results to our study could be obtained using cheaper, screen-based simulation linked with debriefing and whether improved immediate recall translates into improved long-term knowledge retention.

## Conclusion

In conclusion our study demonstrates that SIM was superior to PBL for knowledge acquisition in pre-clinical medical students studying hypertension and COPD. Given the limited amount of educational time in the pre-clinical years, more work remains to be done to determine which of these modalities (or whether a combination of both) is best for teaching numerous physiologic, pathophysiologic and pharmacologic concepts, and whether these benefits of knowledge acquisition lead to improvement in performance in the clinical years and beyond.

## Take Home Messages

• Factual learning can be achieved through both high-fidelity simulation and problem-based learning
• Simulation-based education may be superior to problem-based learning for factual acquisition

## Notes On Contributors

**Tyler B. Larsen,** MD is a hospitalist in the Department of Medicine at the West Los Angeles VA Medical Center and a Clinical Instructor of Medicine at the David Geffen School of Medicine at UCLA.

**Nicholas J. Jackson**, MPH, PhD, is an Assistant Professor in the Department of Medicine Statistics Core at the David Geffen School of Medicine at UCLA. He holds an MPH in biostatistics and a PhD in quantitative psychology.

**Jason Napolitano**, MD is an Associate Clinical Professor and an Assistant Dean for Student Affairs at the David Geffen School of Medicine at UCLA. He is Co-Director of a course on cardiovascular, pulmonary and renal pathophysiology for second year medical students which uses high-fidelity simulation during several teaching sessions.

## References

[ref1] Alluri R. K. Tsing P. Lee E. and Napolitano J. (2016) A randomized controlled trial of high-fidelity simulation versus lecture-based education in preclinical medical students. Medical Teacher. 38(4), pp.404–409. 10.3109/0142159X.2015.1031734 25897707

[ref2] Barrows H. S. and Tamblyn R. M. (1980) Problem-Based Learning: An Approach to Medical Education. New York, NY: Springer Publishing Company.

[ref3] Barsuk J. H. Cohen E. R. Caprio T. McGaghie W. C. (2012) Simulation-Based Education with Mastery Learning Improves Residents. Lumbar Puncture Skills’, Neurology. 79(2), pp.132–137. 10.1212/WNL.0b013e31825dd39d 22675080 PMC3390539

[ref4] Bearman M. O’Brien R. Anthony A. Civil I. (2012) Learning Surgical Communication, Leadership and Teamwork Through Simulation. Journal of Surgical Education. 69(2), pp.201–207. 10.1016/j.jsurg.2011.07.014 22365866

[ref5] Cohen E. R. Barsuk J. H. Moazed F. Caprio T. (2013) Making July Safer: Simulation-based Mastery Learning During Intern Boot Camp. Academic Medicine. 88(2), pp.233–239. 10.1097/ACM.0b013e31827bfc0a 23269294

[ref6] Harris D. M. Ryan K. and Rabuck C. (2012) Using a High-Fidelity Patient Simulator with First-Year Medical Students to Facilitate Learning of Cardiovascular Function Curves. American Journal of Physiology - Advances in Physiology Education. 36(3), pp.213–219. 10.1152/advan.00058.2012 22952260

[ref7] Hartling L. Spooner C. Tjosvold L. and Oswald A. (2010) Problem-Based Learning in Pre-Clinical Medical Education: 22 Years of Outcome Research. Medical Teacher. 32(1), pp.28–35. 10.3109/01421590903200789 20095771

[ref8] Issenberg S. B. McGaghie W. C. Hart I. R. Mayer J. W. (1999) Simulation Technology for Health Care Professional Skills Training and Assessment. Journal of the American Medical Association. 282(9), pp.861–866. 10.1001/jama.282.9.861 10478693

[ref9] Issenberg S. B. McGaghie W. C. Petrusa E. R. Gordon D. L. (2005) Features and Uses of High-Fidelity Medical Simulations That Lead to Effective Learning: A BEME Systematic Review. Medical Teacher. 27(1), pp.10–28. 10.1080/01421590500046924 16147767

[ref10] Kerr B. Hawkins T. L. A. Herman R. Barnes S. (2013) Feasibility of Scenario-Based Simulation Training Versus Traditional Workshops in Continuing Medical Education: A Randomized Controlled Trial. Medical Education Online. 18(1), pp.1–7. 10.3402/meo.v18i0.21312 PMC3717090 23870304

[ref11] Kinkade S. (2005) A Snapshot of the Status of Problem-Based Learning in U. S. Medical Schools, 2003-04. Academic Medicine. 80(3), pp.300–301. 10.1097/00001888-200503000-00021 15734817

[ref12] Ko P. Y. Scott J. M. Mihai A. and Grant W. D. (2011) Comparison of a Modified Longitudinal Simulation-Based Advanced Cardiovascular Life Support to a Traditional Advanced Cardiovascular Life Support Curriculum in Third-Year Medical Students. Teaching and Learning in Medicine. 23(4), pp.324–330. 10.1080/10401334.2011.611763 22004316

[ref13] Koh G. C. H. Khoo H. E. Wong M. L. and Koh D. (2008) The Effects of Problem-Based Learning During Medical School on Physician Competency: A Systematic Review. CMAJ. 178(1), pp.34–41. 10.1503/cmaj.070565 18166729 PMC2151117

[ref14] Littlewood K. E. Shilling A. M. Stemland C. J. Wright E. B. (2013) High-fidelity simulation is superior to case-based discussion in teaching the management of shock. Medical Teacher. 35(3). 10.3109/0142159X.2012.733043 23126242

[ref15] Lopreiato J. O. and Sawyer T. (2015) Simulation-based medical education in pediatrics. Academic Pediatrics. Elsevier Ltd. 15(2), pp.134–142. 10.1016/j.acap.2014.10.010 25748973

[ref16] McGaghie W. C. Issenberg S. B. Cohen E. R. Barsuk J. H. (2011) Does simulation-based medical education with deliberate practice yield better results than traditional clinical education? A meta-analytic comparative review of the evidence. Academic Medicine. 86(6), pp.706–711. 10.1097/ACM.0b013e318217e119 21512370 PMC3102783

[ref17] Neville A. J. (2009) Problem-based learning and medical education forty years on: A review of its effects on knowledge and clinical performance. Medical Principles and Practice. 18(1), pp.1–9. 10.1159/000163038 19060483

[ref18] Neville A. J. and Norman G. R. (2007) PBL in the Undergraduate MD Program at McMaster University: Three Iterations in Three Decades. Academic Medicine. 82(4), pp.370–374. 10.1097/ACM.0b013e318033385d 17414193

[ref19] Norman G. (2012) Medical education: past, present and future. Perspectives on Medical Education. 1(1), pp.6–14. 10.1007/s40037-012-0002-7 23316454 PMC3540368

[ref20] Okuda Y. Bryson E. O. DeMaria S. Jacobson L. (2009) The utility of simulation in medical education: What is the evidence? Mount Sinai Journal of Medicine.pp.330–343. 10.1002/msj.20127 19642147

[ref21] Poulton T. Ellaway R. H. Round J. Jivram T. (2014) Exploring the efficacy of replacing linear paper-based patient cases in problem-based learning with dynamic web-based virtual patients: Randomized controlled trial. Journal of Medical Internet Research. 16(11), pp.1–10. 10.2196/jmir.3748 PMC4259985 25373314

[ref22] Schmidt E. Goldhaber-Fiebert S. N. Ho L. A. and McDonald K. M. (2013) Simulation exercises as a patient safety strategy: A systematic review. Annals of Internal Medicine. 158(5 PART 2), pp.426–432. 10.7326/0003-4819-158-5-201303051-00010 23460100

[ref23] Schwartz L. R. Fernandez R. Kouyoumjian S. R. Jones K. A. (2007) A Randomized Comparison Trial of Case-based Learning versus Human Patient Simulation in Medical Student Education. Academic Emergency Medicine. 14(2), pp.130–137. 10.1197/j.aem.2006.09.052 17267529

[ref24] Seybert A. L. Smithburger P. L. Kobulinsky L. R. and Kane-Gill S. L. (2012) Simulation-based learning versus problem-based learning in an acute care pharmacotherapy course. Simulation in Healthcare. 7(3), pp.162–165. 10.1097/SIH.0b013e31825159e3 22653560

[ref25] Shin S. Park J. H. and Kim J. H. (2015) Effectiveness of patient simulation in nursing education: Meta-analysis. Nurse Education Today. Elsevier Ltd. 35(1), pp.176–182. 10.1016/j.nedt.2014.09.009 25459172

[ref26] Singer B. D. Corbridge T. C. Schroedl C. J. Wilcox J. E. (2013) First-year residents outperform third-year residents after simulation-based education in critical care medicine. Simulation in Healthcare. 8(2), pp.67–71. 10.1097/SIH.0b013e31827744f2 23222546 PMC3610783

[ref27] Smith K. V. Witt J. Klaassen J. A. Zimmerman C. (2012) High-fidelity simulation and legal/ethical concepts: A transformational learning experience. Nursing Ethics. 19(3), pp.390–398. 10.1177/0969733011423559 22323395

[ref28] Steadman R. H. Coates W. C. Yue M. H. Matevosian R. (2006) Simulation-based training is superior to problem-based learning for the acquisition of critical assessment and management skills. Critical Care Medicine. 34(1), pp.151–157. 10.1097/01.CCM.0000190619.42013.94 16374169

[ref29] Ten-Eyck R. P. Tews M. and Ballester J. M. (2009) Improved Medical Student Satisfaction and Test Performance With a Simulation-Based Emergency Medicine Curriculum: A Randomized Controlled Trial. Annals of Emergency Medicine. 54(5), pp.684–691. 10.1016/j.annemergmed.2009.03.025 19394113

[ref30] Wenk Manuel Waurick R. Schotes D. Wenk Melanie (2009) Simulation-based medical education is no better than problem-based discussions and induces misjudgment in self-assessment. Advances in Health Sciences Education. 14(2), pp.159–171. 10.1007/s10459-008-9098-2 18214702

