# Supplementary material for: A Comparison of Simulation-Based Education and Problem-Based Learning in Pre-Clinical Medical Undergraduates
Source: MedEdPublish (2016). 2020 Aug 19;9:172. [Version 1] doi: 10.15694/mep.2020.000172.1 (PMC10702657; doi:10.15694/mep.2020.000172.1)
Supplement: Peer review history

# Reviewer report: Reviewer response for version 1

**DOI:** 10.21956/mep.20069.r30829

**Published:** 2020-11-11

**Authors:** Ken Masters

**Affiliations:** 1 Sultan Qaboos University

This review has been migrated. The reviewer awarded 5 stars out of 5 An interesting randomized, cross-over study comparing simulation-based education and PBL in pre-clinical medical undergraduates.Overall, the study has been very well-conducted and very well-presented in the paper. In such a study, there will always be limitations, and the most important of these have been noted and addressed by the authors (that the strength of PBL is not really in factually-based work). The authors have also noted that simulation-based education can be expensive. There is also the fact that individual PBL facilitators will impact on students’ result, but, given that this study was spread across the entire cohort, this is unlikely to have been an influencing factor.Later, follow-up studies will be able to add more information to the results, but, for now, this study certainly indicates that, for factual work, sim-based education can deliver results are at least, and possibly better, than PBL. I am sure that the paper will be useful evidence for medical schools considering broadening their teaching in this way.

Reviewer Expertise:

NA

No decision status is available

# Reviewer report: Reviewer response for version 1

**DOI:** 10.21956/mep.20069.r30828

**Published:** 2020-08-22

**Authors:** John Cookson

**Affiliations:** 1 University of Worcester

This review has been migrated. The reviewer awarded 5 stars out of 5 Thanks, an interesting study.Many people have tried moving away from a paper-based PBL ‘case’ to video or SPs, so perhaps this paper aligns with that. As the authors state, PBL is not just about learning facts, it’s as much about finding out what you don’t know. It would be interesting to know what the students were thinking as they went through the scenario. A de-brief which included a ‘think-aloud’ session might be revealing. Notwithstanding that the Simulation was in a ‘safe-space’ it probably gets the adrenalin flowing more than the average PBL session. Is it that that makes the facts ‘sticker’? What if the adrenalin goes too high-one would anticipate less learning perhaps? The resources used are interesting, particularly staff time. If I interpret it correctly the PBL time lasted 4 hours but the Simulation 20 mins. In these comparisons it seems important to look for efficiency of learning; how much learning is acquired per unit time. On the face of it Simulation was not only better but highly efficient. However I’m not sure if that conclusion can be drawn here on the data.

Reviewer Expertise:

NA

No decision status is available

# Reviewer report: Reviewer response for version 1

**DOI:** 10.21956/mep.20069.r30827

**Published:** 2020-08-19

**Authors:** Richard J. Nierenberg

**Affiliations:** 1 Hackensack University Medical Center, Hackensack Meridian Health

This review has been migrated. The reviewer awarded 5 stars out of 5 This is a very interesting and important study in that it compares in a rigorous fashion the learning of key concepts of basic clinical entities between two modes of learning, problem based discussion, which is time tested and universally available, and newer SIM based learning which is less widely distributed, requires investment in time, training and material, but nonetheless is shown in this study to improve the ability of learners to master concepts required, at a minimum, to pass written examinations on the subject matter.The authors conduct a crossover study which demonstrates the superiority of SIM based learning in either order of presentation when compared to PBL sessions.Although one might have intuitively thought that SIM based learning, while superior for hands on, so called "practical skills", would not show superiority for a more cognitive -question answering assessment.The authors provide a plausible explanation for why that may be so, in that being placed into a (simulated) clinical situation evokes the emotional connection with the material, the more or less visceral understanding of its clinical importance, which makes the material stick.The authors point out that SIM technology is not universally available.I wonder whether the good bedside teacher could formulate questions to learners in order to take advantage of this now known beneficial effect of a SIM model. Perhaps the creation in the learners mind and in real time of possible realistic imaginary clinical scenarios, having the student imagine, for example, during the case presentation of a chest pain patient, that the patient is dropping their blood pressure, skipping beats, having more chest pain, and the like. In that manner, pending creation of a SIM program, teachers can utilize the knowledge, confirmed in this study, that SIM improves learning, even in pre-clinical years and for basic pathophysiologic concepts.A very intriguing and interesting study!

Reviewer Expertise:

NA

No decision status is available
